# Development of an interdisciplinary consensus statement for assessing fitness for work at heights in the South African construction industry: a virtual Modified Nominal Group Technique study

**DOI:** 10.1186/s12995-026-00500-0

**Published:** 2026-02-26

**Authors:** Lyndsey Swart, Tania Buys, Nicolaas Claassen

**Affiliations:** 1 Department of Occupational Therapy, School of Health Care Sciences, University of Pretoria, Pretoria, South Africa; 2 Division for Environmental and Occupational Health, School of Health Systems and Public Health, University of Pretoria, Pretoria, South Africa

**Keywords:** Fitness for work, Work at heights, Construction safety, Occupational health, Consensus development, Nominal Group Technique

## Abstract

**Background:**

Falls from heights are a leading cause of occupational injury and death globally, with construction workers disproportionately affected. In South Africa, employers must ensure that workers performing fall-risk tasks are certified as fit to work at heights, yet regulations provide little guidance on how such assessments should be conducted. Within a broader two-phase research project undertaken by the authors, Phase 1 comprised a scoping review that identified limited peer-reviewed evidence and a lack of standardised frameworks for assessing fitness for work at heights, followed by a qualitative study that found inconsistent, predominantly medicalised assessment practices that inadequately reflect job-specific risks and demands. In response, a draft interdisciplinary consensus statement was developed. This study reports Phase 2, a structured expert consensus process undertaken to systematically revise and consolidate the draft consensus statement.

**Methods:**

A virtual Modified Nominal Group Technique was conducted with six experts from occupational medicine, occupational health nursing, occupational therapy, and construction health and safety. Participants reviewed the draft consensus statement prior to a facilitated online discussion, followed by an anonymous post-session rating survey. Quantitative ratings were analysed using medians and interquartile ranges against predefined consensus criteria, while qualitative data from transcripts, field notes, and participant annotations were analysed using directed qualitative content analysis.

**Results:**

Consensus was achieved on 20 of 27 items, indicating strong support for the draft statement’s overall structure and intent. Items not reaching consensus mainly concerned definitional clarity, occupational risk-exposure profiling, and follow-up procedures. Revisions focused on clarifying terminology; strengthening guidance on occupational risk exposure and worker–job specification; recognising behavioural and psychosocial factors alongside physical, cognitive, and environmental considerations; and introducing the concept of a *competent, registered and authorised person*.

**Conclusions:**

This study presents an interdisciplinary consensus statement, developed through expert consensus, providing a principles- and process-based framework for assessing fitness for work at heights. It promotes consistent, transparent, job-specific, risk-based fitness assessments beyond generic medical certification. Future efforts should focus on translating this framework into practical tools and evaluating its feasibility across various construction settings.

**Supplementary Information:**

The online version contains supplementary material available at 10.1186/s12995-026-00500-0.

## Background

Falls from heights remain a leading cause of occupational injury and death worldwide [1–3], with the construction industry consistently identified as a high-risk sector [4–8]. In South Africa, employers are required to ensure that employees performing construction work at heights hold a valid medical certificate of fitness, specific to the construction work to be performed, and issued by a practitioner designated in the Occupational Health and Safety Act (1993) and the Construction Regulations (2014) as an *occupational health practitioner*, using the prescribed Annexure 3 format [9, 10]. While this regulatory framework mandates certification, it does not specify how fitness for work at heights (FFWAH) should be evaluated or what constitutes sufficient evidence of fitness in relation to job-specific risks and demands.

In the absence of clear guidance, FFWAH assessment practices vary substantially. To address this gap, the authors undertook a comprehensive two-phase research project to support the development of an interdisciplinary consensus statement for assessing FFWAH in the South African construction industry. Phase 1 focused on evidence synthesis through a scoping review of international and South African peer-reviewed and grey literature, together with semi-structured interviews with an interdisciplinary group of experts involved in FFWAH assessment. These activities informed the development of a draft interdisciplinary consensus statement. In this study, *interdisciplinary* denotes the integration of perspectives from occupational medical practitioners, occupational health nurses, occupational therapists, and construction health and safety professionals, rather than reliance on a single disciplinary or profession-specific approach.

The scoping review mapped existing approaches to assessing FFWAH [11, 12] and identified a marked lack of standardised FFWAH assessment frameworks, tools, or agreed fitness thresholds [13–16], alongside considerable variation in assessment practices among health professionals [17]. Existing approaches were found to rely predominantly on baseline medical examinations that prioritise general health status, with limited consideration of the physical, cognitive, behavioural, and contextual capacities required for safe work at heights [17–19].

The semi-structured interviews further explored these gaps and revealed a strong convergence of expert perspectives that FFWAH assessment should be regarded as a cross-disciplinary determination rather than solely a medical judgement [20–22]. Participants highlighted that, despite regulatory emphasis on job-related fitness certification, current practices often fail to systematically incorporate occupational risk exposure, task-specific job demands, and changing work conditions [23–25]. This misalignment weakens the consistency and defensibility of FFWAH determinations, increasing uncertainty and risk for employers, workers, and practitioners [26, 27].

Collectively, Phase 1 findings conceptualised FFWAH as a dynamic interaction between individual worker capacities, task-specific job demands, and contextual and environmental conditions [12]. This conceptualisation aligns with a biopsychosocial approach [28] and the International Classification of Functioning, Disability and Health (ICF) [29], and underscored the need for clearer definitions, more structured job-specific assessment processes, more nuanced fitness outcomes, and stronger interdisciplinary collaboration.

These insights informed the development of a draft interdisciplinary consensus statement to articulate shared principles and core process elements to guide FFWAH assessment. Phase 1 demonstrated that, in the absence of agreed foundational principles and assessment processes, specifying detailed protocols, tools, or item-level criteria would be premature. Accordingly, the draft consensus statement adopted a principles- and process-first approach, establishing a common conceptual and procedural foundation to support consistent and defensible FFWAH decision-making and to inform subsequent development of context-appropriate assessment tools and instruments.

The present study reports on Phase 2 of the broader research project, which aimed to revise and consolidate the draft consensus statement through a structured expert consensus process. Using a virtual Modified Nominal Group Technique (vMNGT), this phase focused on strengthening foundational principles and assessment processes, clarifying areas of ambiguity, and achieving expert consensus where disagreement existed. The study did not seek to develop or validate specific assessment tools, tests, or fitness thresholds.

## Methods

This study draws on the ACCORD reporting guidelines (ACcurate COnsensus Reporting Documents) [30] to enhance transparency and completeness in documenting the vMNGT and consensus development process (Additional file 1).

### Study design

The study was grounded in a pragmatic paradigm, aligned with its practical, solution-focused aim of revising and consolidating a draft consensus statement [31]. A vMNGT was chosen to gather geographically dispersed expert input in a structured manner to facilitate consensus development. The Nominal Group Technique is a recognised consensus-building method in healthcare research, particularly valuable when peer-reviewed evidence is scarce and expert judgment, structured interaction, and deliberation are required to inform practice or policy [32–35]. The vMNGT retained the core features of a traditional NGT, including structured discussion and immediate post-discussion rating, but was modified through virtual delivery and pre-session circulation of the draft consensus statement.

### Participant recruitment and selection

Six interdisciplinary expert participants were purposively sampled to achieve professional diversity and direct involvement in FFWAH assessment and/or management [36–38]. Inclusion criteria comprised: (i) occupational medical practitioners and occupational health nurses with experience conducting Annexure 3 medical examinations; (ii) occupational therapists with experience in functional assessment related to work at heights; and (iii) construction health and safety practitioners involved in managing health and fitness requirements for workers at heights on construction sites.

Participants were recruited through professional associations, including the South African Society of Occupational Medicine (SASOM), the South African Society of Occupational Health Nursing (SASOHN), the South African Council for the Project and Construction Management Professions (SACPCMP), and the Occupational Therapy Association of South Africa (OTASA), supplemented by snowball sampling. Four participants had previously contributed to Phase 1 of the broader study, while two additional experts were identified through peer referral. Participants completed a standardised demographic questionnaire (Additional file 2). Eight experts meeting the inclusion criteria were initially contacted by telephone, followed by email correspondence containing study information and consent documentation. Six experts agreed to participate; two declined due to availability constraints.

### Study procedure

One week before the vMNGT session, participants received an information pack containing the draft consensus statement, an outline of the vMNGT process, and information on the session’s purpose and structure to support informed participation. The online session was conducted via Zoom and facilitated by the three co-authors, each fulfilling predefined roles to promote structure and neutrality: LS introduced the process and administered the consensus survey; TB moderated the discussion; and NC provided technical support and real-time documentation. The session was audio-recorded and automatically transcribed via Zoom.

Immediately following the facilitated discussion, participants independently completed an anonymous, structured consensus survey administered via Qualtrics. Each participant accessed the survey individually, rated all sections of the draft consensus statement against predefined criteria, and could not view other participants’ responses.

**Fig. 1:**
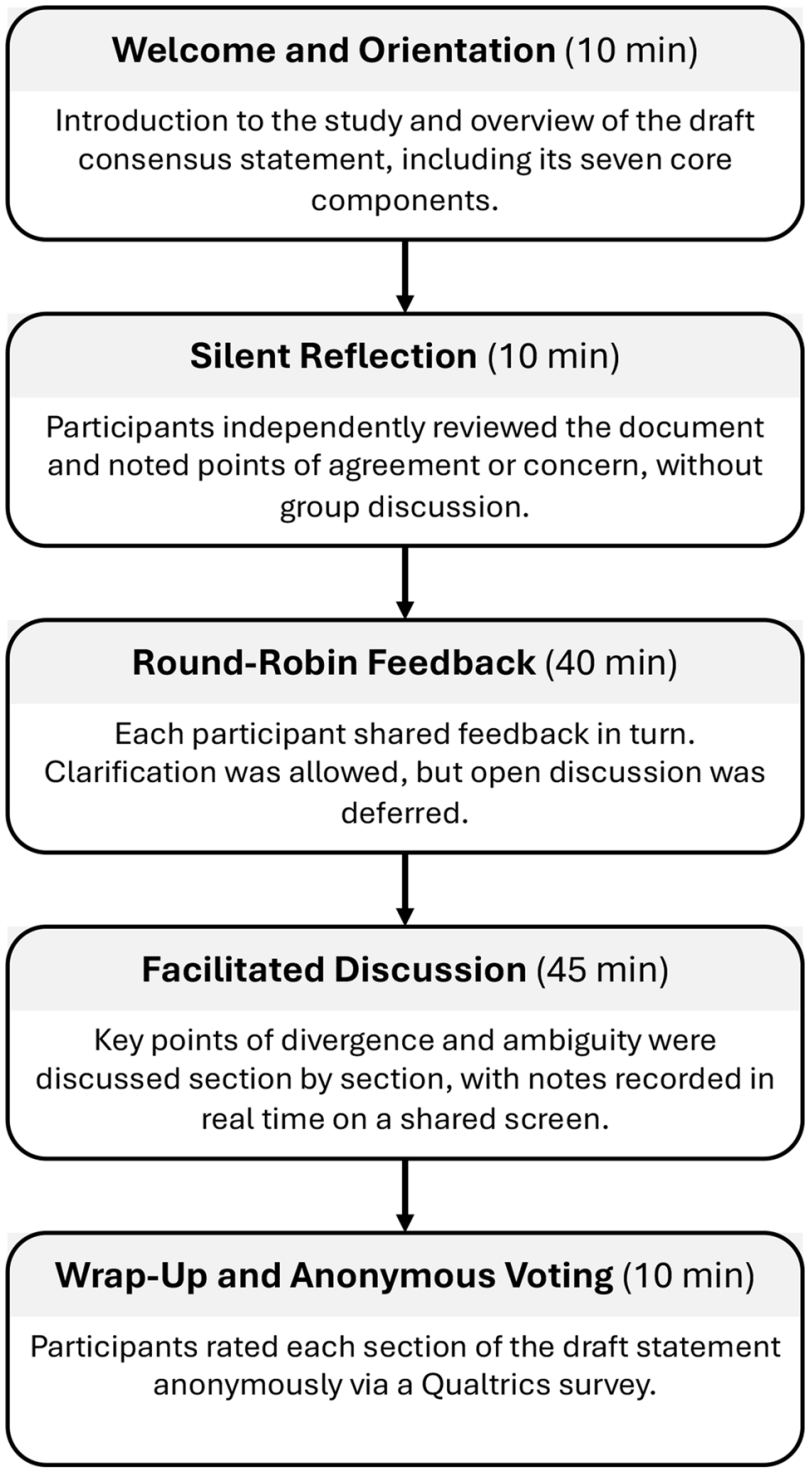
Virtual Modified Nominal Group Technique process for consensus development

The vMNGT followed a five-step procedure adapted from Potter et al. [32] (Fig. 1), incorporating individual review of the draft consensus statement, structured feedback, facilitated discussion, and consensus evaluation. A pilot session involving one participant was conducted to test technical functionality, including Zoom access, audio quality, document sharing, and the Qualtrics survey workflow. No study content was discussed during the pilot.

### Data collection and analysis

#### Quantitative data

A structured, purpose-designed consensus survey was used to evaluate the draft consensus statement following the vMNGT. Survey items were developed by aligning each part of the draft statement with established evaluation criteria: clarity, relevance, practicality, robustness of the framework, and the credibility of the consensus development process. The draft survey was reviewed by the research team for content alignment and clarity, and it was tested during the technical pilot session to check platform functionality and response flow. Table 1 summarises the criteria applied to each section. The complete survey instrument is provided in Additional file 3. Participants rated each section on a 5-point Likert scale (1 = strongly disagree to 5 = strongly agree), resulting in 27 ratings across 10 sections.

**Table 1:** Sections of the draft consensus statement and corresponding evaluation criteria

Section		Criteria rated per section							
		Purpose, scope & relevance	Credibility of CS development	Robust framework	Clarity	Relevance	Practicality	Summary	Recommendations
1	Introduction	X	X						
2	Key definitions				X	X			
3	FFWAH 5-Step procedure			X	X		X		
4	Occupational risk exposure profile (OREP)				X	X	X		
5	Worker-job specification (WJS)				X	X	X		
6	FFWAH assessment approach				X	X	X		
7	Annexure 3 medical certificate of fitness				X	X	X		
8	Follow-up of workers with limitations or restrictions				X	X	X		
9	Legal, ethical, and regulatory compliance				X	X	X		
10	Conclusion							X	X

CS = consensus statement; FFWAH = Fitness for work at heights

Given the ordinal nature of single-item Likert data and the small, purposively sampled group (*n* = 6), descriptive statistics were used [39–43]. Medians were calculated to summarise central tendency, and interquartile ranges (IQRs) were used to indicate dispersion and the strength of agreement. IQRs were calculated using the Tukey method in Stata v18.0 BE (StataCorp LLC, College Station, TX, USA).

Consensus thresholds were established a priori (Table 2): consensus was defined as a median ≥ 4 and IQR ≤ 1; general support without consensus as a median ≥ 4 and IQR > 1; and no consensus as a median < 4. These thresholds align with established guidance for consensus studies and are consistent with NGT-based scoring/ranking approaches, where higher median ratings and narrower IQRs reflect stronger central agreement and less dispersion in panel ratings [44–46]. Inferential statistics were not applied, as the aim was to examine the convergence of expert opinion rather than to generalise findings.

**Table 2:** Consensus interpretation thresholds

Median & IQR	Interpretation	Rationale
Median ≥4 and IQR ≤1	Consensus	Clear agreement among participants with strong convergence of views; high support and low variability.
Median ≥4 and IQR > 1	General support – without consensus	Indicates that most participants supported the statement, but responses were insufficiently convergent to meet consensus criteria.
Median < 4	No consensus	Reflects low overall support and/or wide variability in participant ratings, indicating that agreement was not reached on the statement.

#### Qualitative data

Qualitative data included the Zoom transcript, field notes, and shared screen annotations. The transcript was reviewed against the audio recording to correct transcription errors and remove filler words and timestamps. A directed qualitative content analysis [47] was undertaken, using the draft consensus statement as an a priori framework. Data were examined section by section to identify areas of agreement, points of concern or disagreement, and proposed revisions. This framework-aligned approach enabled systematic integration of expert input and informed targeted amendments to the draft consensus statement.

During the revision process, ambiguities were identified regarding statutory terminology and the recognition and scope of professional roles under the Occupational Health and Safety Act and the Construction Regulations. To ensure legally defensible and contextually appropriate wording, the research team sought independent legal consultation, which informed the adoption of the term *competent, registered, and authorised person* in the revised consensus statement. In addition to participant-generated revisions, the lead researcher made limited editorial adjustments to improve clarity, coherence, and terminological consistency. These edits were documented separately to distinguish them from expert-driven contributions.

### Ethical considerations

Ethical approval was obtained from the University of Pretoria, Faculty of Health Sciences Research Ethics Committee (Ref: 486/2021). All participants gave written informed consent to participate in the vMNGT session and subsequent communications. Confidentiality was preserved through the use of participant codes (P1–P6), and identifying information was removed from transcripts and reported materials.

### Researcher reflexivity

Researcher reflexivity was explicitly addressed to manage the potential influence of the researchers’ professional backgrounds and any prior relationships with participants. All participants were senior practitioners, and there were no formal academic, organisational, supervisory, or employment relationships between the researchers and participants. Aside from one participant (OT-1), who was a professional colleague known to LS and TB in a collegial, non-hierarchical capacity, none of the authors had any previous professional or personal relationship with any participant, and no author held evaluative authority over any participant.

As the lead investigator of both phases of the broader research project and developer of the underlying conceptual framework, LS recognised the potential for this level of involvement to influence deliberations and therefore did not moderate the vMNGT discussion. Reflexivity was further supported through ongoing self-monitoring during data analysis and structured peer debriefing with co-authors TB and NC to challenge assumptions, improve transparency, and support analytic credibility [48].

## Results

### Participant characteristics

Six experts participated: two occupational medical practitioners, two occupational health nurses, one occupational therapist, and one construction health and safety practitioner. All worked in the private sector across four South African provinces: Eastern Cape, Western Cape, Gauteng, and KwaZulu-Natal. Five were self-employed, while one was employed by a medium-sized private organisation. All had over 10 years of professional experience (four had more than 20 years). All held at least a bachelor’s degree; four had master’s degrees (including one in law), and one held a PhD.

### Quantitative consensus ratings

Table 3 summarises the quantitative consensus ratings across the ten sections of the draft consensus statement, evaluated against predefined criteria. Overall, consensus was reached on 20 of the 27 ratings (74%), indicating substantial agreement among participants across six sections of the draft consensus statement: the introduction, the WJS, FFWAH assessment approach, Annexure 3 medical certificate of fitness, legal, ethical and regulatory compliance, and the conclusion.

**Table 3:** Post-session consensus ratings for the draft consensus statement

Rating	Section	Criterion	Median	IQR	Consensus Interpretation
1	Introduction	Intro – Purpose, scope & relevance	4	0.75	Consensus
2	Introduction	Intro – Credibility of CS development	4	0.75	Consensus
3	Key definitions	Definitions – Clarity	3	2.75	No consensus
4	Key definitions	Definitions – Relevance	4	0.75	Consensus
5	3.1	5-Step procedure – Clarity	4.5	1	Consensus
6	3.1	5-Step procedure – Robust framework	4	0	Consensus
7	3.1	5-Step procedure – Practicality	4	1.5	General support – without consensus
8	3.2	OREP – Clarity	4	2.25	General support – without consensus
9	3.2	OREP – Relevance	4.5	1	Consensus
10	3.2	OREP – Practicality	4	2.25	General support – without consensus
11	3.3	WJS – Clarity	4.5	1	Consensus
12	3.3	WJS – Relevance	5	0.75	Consensus
13	3.3	WJS – Practicality	4.5	1	Consensus
14	3.4	Assessment approach – Clarity	4	0.75	Consensus
15	3.4	Assessment approach – Relevance	4	0.75	Consensus
16	3.4	Assessment approach – Practicality	4.5	1	Consensus
17	3.5	Annexure 3 – Clarity	4	0	Consensus
18	3.5	Annexure 3 – Relevance	4	0.75	Consensus
19	3.5	Annexure 3 – Practicality	4	0.75	Consensus
20	3.6	Follow-up – Clarity	3.0	2	No consensus
21	3.6	Follow-up – Relevance	4.5	2.5	General support – without consensus
22	3.6	Follow-up – Practicality	2.0	1.5	No consensus
23	3.7	Compliance – Clarity	4	0.75	Consensus
24	3.7	Compliance – Relevance	5	0.75	Consensus
25	3.7	Compliance – Practicality	4.5	1	Consensus
26	Conclusion	Conclusion – Summary	4	0.75	Consensus
27	Conclusion	Conclusion – Recommendations	4	0.75	Consensus

CS = Consensus statement; OREP = Occupational risk exposure profile; WJS = Worker-job specification; FFWAH = Fitness for work at heights

Seven ratings (26%) did not meet the predefined consensus criteria and were distributed across four sections: key definitions, the five-step procedure, the occupational risk exposure profile (OREP), and follow-up of workers. Of these, four ratings achieved a median score of ≥ 4 but exceeded the IQR threshold, indicating general support without clear consensus. These concerned the practicality of the five-step procedure, the clarity and practicality of the OREP, and the relevance of follow-up. In these cases, participants broadly supported the intent of the sections but differed in how they interpreted or applied the content, resulting in greater variability in responses.

The remaining three ratings did not achieve consensus, with low median scores and wide dispersion, indicating areas requiring significant revision. These concerned the clarity of key definitions and the clarity and practicality of the follow-up process.

Analysis of these non-consensus items revealed two underlying patterns. Firstly, limited clarity and a lack of shared understanding led to differing interpretations, especially concerning key definitions and the OREP. Secondly, concerns about the feasibility of implementation within the operational constraints of the construction industry influenced evaluations of practicality, especially for follow-up processes. One participant consistently rated several items lower than the rest of the panel, with accompanying comments emphasising the need for legal precision, clearer procedural requirements, and explicit delineation of professional roles.

These quantitative patterns guided the subsequent qualitative analysis, which explored the factors underlying agreement and disagreement and how expert input shaped revisions to the draft consensus statement.

### Qualitative outcomes

Qualitative findings are presented by section of the draft consensus statement. For each section, the accompanying table summarises key discussion points, illustrative quotations, and resulting amendments, while the narrative text interprets areas of agreement or disagreement and their implications for FFWAH assessment practice.

#### Introduction

Table 4 summarises expert feedback and the resulting revisions to the introduction. Consensus was reached on its purpose, scope, relevance, and the credibility of the consensus development process.

**Table 4:** Revisions to Introduction based on expert feedback

Salient Discussion Points	Illustrative Quotes	Revisions Made
The word “uncertainty” in the first paragraph is unclear.	“… remove the word ‘uncertainty’ and restructure the sentence … make a statement that … we don’t have a standardised systematic way of conducting medicals throughout the sector.” [P6]	Reworded to reflect the absence of formal guidance and the resulting inconsistency in practical implementation of FFWAH assessments.
Indicate that the consensus statement addresses the assessment of fitness for job, not fitness for duty.	“… that clarification [fit for job as opposed to fit for duty] makes it clear.” [P4]	Clarified in the Introduction.
The scope of the consensus statement should be more clearly defined.	“Maybe it needs a preamble … [that outlines] the scope of the document …” [P2]	Clarified scope: fitness for job versus fitness for duty; scope of FFWAH assessments; definition of fall risk.
FFWAH is a holistic assessment that includes psychosocial, behavioural, environmental, and contextual factors in addition to physical ability.	“There are going to be other co-contributors to the assessment than just sheer fall-risk … there are safety factors, there are ergonomic factors, and there are physicality factors ….” [P2] “… we need to be physically and psychologically fit to work at heights, not just physically fit.” [P6] “It’s actually the inherent requirements of the job … the operational demands associated with a specific task or role.” [P2]	Linked assessment approach to ICF/biopsychosocial model.
Overlapping professional roles and legal ambiguity regarding FFWAH responsibilities.	“I would like to raise that the document must respect the registered scopes of the different professions.” [P1]	Introduced the term *competent, registered, and authorised person*.

FFWAH = Fitness for work at heights; ICF = International Classification of Functioning, Disability and Health

Qualitative discussion focused on sharpening the scope of the consensus statement rather than introducing new content. Participants emphasised the need to clearly distinguish fitness for job, addressed through the FFWAH evaluation and Annexure 3 certification, from fitness for duty, which was positioned as an ongoing, site-based safety responsibility. The introduction was further revised to explicitly reflect the multifaceted nature of FFWAH assessment, encompassing physical, cognitive, behavioural, environmental, and contextual factors.

Participants also highlighted regulatory ambiguity and overlapping professional roles in FFWAH assessment. To acknowledge the need for appropriate competence without prescriptively assigning responsibility to specific professions, the term *competent, registered, and authorised person* was introduced to support consistent interdisciplinary interpretation while respecting existing scopes of practice.

#### Key definitions

Table 5 presents expert feedback and revisions relating to the key definitions proposed for the consensus statement. Consensus was achieved on relevance, but not on clarity.

**Table 5:** Revisions to key definitions

Salient Discussion Points	Illustrative Quotes	Revisions Made
*Definitions of work at heights, occupational risk exposure profilec (OREP)*, and *fit for job* lacked clarity.	“… the term OREP, perhaps is being misunderstood … the OREP has to define everything about [what] the person needs for the job.” [P2]	Revised definitions for clarity.
Definition of *work at heights* omitted hazard element (*fall risk*).	“I don’t like the definition of falling … it doesn’t include our current definition of the hazard … which is defined as the fall risk …” [P1]	Incorporated Construction Regulation definition of *fall risk*.
*Fit for job* should reflect full job scope and risks as defined in the WJS and OREP.	“The conversation needs to look at … the full risk [of working at height]” [P2] “… the Worker-job specification must establish the standards for the testing and the reference standards for risk acceptance.” [P1]	Expanded definition to integrate WJS and OREP.
*Fit for duty* is an ongoing safety issue to be monitored by employers.	“[Supervisors and safety officers] really are the ones with the … eyes on the employee in the working environment.” [P4]	Expanded definition to include employer monitoring role.
Need for legal clarity and respect for professional scopes of practice.	“[The] scope of practice is a vital thing that must be referred to.” [P5] “… this document [must] respect the current registration of professionals …” [P1]	Defined the term *competent, registered, and authorised person*.
Behavioural/psychological risk factors absent.	“So when my psychological profile comes back, it will show me … [the worker] has a tendency to high-risk behaviours …” [P6]	Added behavioural risks to OREP and WJS.
Functional job demands analysis should be offered when OREPS do not sufficiently capture functional job demands.	“… when the OREPs are not sufficiently captured, a task-specific demands and detailed functional job demands analysis [may be] required.” [P5]	Definition of *functional job demands analysis* (FJDA) included

OREP = Occupational risk exposure profile; WJS = Worker-job specification

Discussion revealed that inconsistent interpretation of foundational terms—particularly work at heights, OREP, and fit for job—contributed to variability in FFWAH assessment practice across disciplines. Revisions, therefore, prioritised definitional precision, alignment with regulatory language, and clearer articulation of how these concepts function within the FFWAH assessment process. These changes aimed to establish a shared and consistent terminology, regarded as essential for the development and application of fundamental principles for FFWAH assessment.

#### Five-step procedure

Table 6 (a) summarises expert feedback and revisions relating to the five-step FFWAH assessment procedure. Consensus was achieved on clarity and robustness, but not on practicality.

**Table 6:** Revisions to (**a**) Five-step procedure and (**b**) Occupational risk exposure profile (OREP)

Salient Discussion Points	Illustrative Quotes	Revisions Made
**(a) Five-step procedure**		
Legal requirement for certificate of fitness not correctly stated in Step 4.	“… issue the construction regulation medical certificate of fitness in the form of Annexure 3. Not [just] Annexure 3.” [P1]	Wording corrected to specify “construction regulation medical certificate of fitness in *the form of* Annexure 3.”
**(b) OREP**		
Need for competence in developing the OREP.	“… a suitably qualified profession [to analyse job demands] is listed, such as an occupational therapist. But … the scope of profession of an occupational medical practitioner, and certainly of an occupational medical specialist, falls in there, too.” [P1]	Inclusion of the term *competent, registered, and authorised person* in relation to OREP development.
OREP definition requires clarification of its purpose, the components it should include, and its intended use within the FFWAH assessment process.	“[The OREP is] not just about the exposures to which the person may be exposed. It’s about … the operational demands associated with a specific task or role. So it includes psychological …” [P2] “Is this person going to be a high-risk behaviour based individual and that’s what I want to see clearly worded in theOccupational risk exposure profile …” [P6]	Definition expanded accordingly.

OREP = Occupational risk exposure profile

Participants broadly supported the procedure’s structure and sequence. Concerns about practicality mainly focused on the challenge of implementing all steps consistently within the operational limits of the construction industry, including time pressures, the varying availability of job-specific information, and resource constraints in smaller organisations. Revisions were limited to minor terminological clarification in Step 4 to ensure precise alignment with the wording of the Construction Regulations, while maintaining the overall procedural structure.

#### Occupational risk exposure profile (OREP)

Table 6(b) presents expert feedback and revisions relating to the OREP. Consensus was achieved on relevance, but not on clarity or practicality.

Discussion indicated agreement that the OREP is essential for grounding FFWAH assessments in the specific risks associated with work at height. However, variability in ratings reflected differing interpretations of the OREP’s purpose, content, and intended use across disciplines. Revisions focused on clarifying the role of the OREP within the assessment process, expanding its scope beyond physical hazards to include cognitive, behavioural, and psychosocial risk factors, and specifying that development of the OREP should be undertaken by a *competent, registered, and authorised person*.

#### Worker–job specification (WJS)

Table 7 (a) summarises expert feedback and revisions relating to the WJS. Consensus was achieved on clarity, relevance, and practicality.

**Table 7:** Revisions to (**a**) Worker-job specification (WJS); (**b**) Fitness for work at heights (FFWAH) evaluation; and (**c**) Annexure 3 medical certificate of fitness

Salient Discussion Points	Illustrative Quotes	Revisions Made
**(a) WJS**		
The need for competence in developing the WJS	… whoever is [compiling the WJS] must do it properly …” [P2]	*Competent, registered, and authorised person* included.
The WJS must be comprehensive and support informed decision-making.	… the person who then compiles the worker-job specification must make sure it’s a comprehensive worker-job specification, including all the aspects.” [P2] “It should also include … the reference standard for risk acceptance when that’s relevant.” [P1]	Content of WJS outlined.
**(b) FFWAH evaluation**		
The need for competence in conducting FFWAH evaluations	… so I think that ‘where necessary’ should be taken out. I think if you get to work with a fall risk, it has to be done by a specialist person.” [P1]	*Competent, registered, and authorised person* included.
*Fit with limitations* should also be included in the worker fitness classification.	… you didn’t include all your options … workers who have limitations or restrictions or conditions …” [P1]	Worker fitness classification expanded to include limitations.
**(c) Annexure ** 3 ** certificate**		
A competent person is required to issue the certificate of fitness.	But when it comes to making a decision, whether a person is fit or not, there is a lot that comes to party …” [P3]	*Competent, registered, and authorised person* included.
Annexure 3 does not make provision for the different classifications of fitness.	The Annexure 3 only talks to the fitness of the employee. It doesn’t give us the accommodations that you talk about in 3.4.” [P3]	Requirement to specify restrictions and conditions expanded to include limitations.

OREP = Occupational risk exposure profile; WJS = Worker-job specification

Participants consistently viewed the WJS as central to translating occupational risk exposure into explicit job demands against which worker fitness can be evaluated. Revisions strengthened articulation of required content, emphasised job specificity, the specification of reference standards where relevant, and clarified responsibility for compilation by a competent, registered, and authorised person.

#### FFWAH evaluation

Table 7(b) presents expert feedback and revisions relating to the FFWAH evaluation. Consensus was achieved across all criteria.

Discussion supported a comprehensive evaluation approach incorporating medical, functional, cognitive, behavioural, and psychosocial considerations. Revisions reinforced explicit linkage between evaluation outcomes and the preceding OREP and WJS, clarified professional competence requirements, and revised fitness classifications to include a *fit with limitations* category.

#### Annexure 3 certificate of fitness

Table 7 (c) summarises expert feedback and revisions relating to the Annexure 3 medical certificate of fitness. Consensus was achieved on clarity, relevance, and practicality.

Discussion highlighted the central role of the Annexure 3 certificate as the formal regulatory output of the FFWAH assessment process, while also recognising its limitations in capturing nuanced fitness outcomes. Revisions clarified responsibility for issuing the certificate and strengthened guidance on documenting restrictions, conditions, and limitations to improve alignment with the assessment process.

#### Follow-up of workers

Table 8(a) presents expert feedback and revisions relating to follow-up of workers following FFWAH assessment. Consensus was not achieved on clarity, relevance, or practicality.

**Table 8:** Revisions to (**a**) Follow-up of workers; and (**b**) Legal, ethical and regulatory compliance

Salient Discussion Points	Illustrative Quotes	Revisions Made
**(a) Follow-up of workers**		
There are many practical challenges to conducting follow-up and medical surveillance in the construction industry.	“When you have your restrictions on your certificate, it’s difficult to get those people back for follow-up.” [P3]	Guidance on follow-up added to the Conclusion, recommending that organisational policy address the process for monitoring workers between FFWAH assessments.
Include all classifications of worker fitness.	“… but you [did not] include the employees who work under conditions.” [P1]	Follow-up section revised to specify workers with conditions, limitations, or restrictions.
Collaboration with the employer optimises worker follow-up. Safety personnel and line managers are often best positioned to observe workers on- site.	“… ensuring that safety personnel and line managers are part of that collaboration … they really are the ones with the eyes on the employee in the working environment.” [P4] “… appropriate management of limitations which include your reasonable accommodations and advice on ongoing monitoring and adjustment or reintegration.” [P5]	Requirement introduced for occupational health professionals to collaborate with employers, safety personnel, and line managers.
**(b) Legal, ethical, and regulatory Compliance**		
No changes proposed.	––	––

Discussion highlighted substantial challenges in implementing follow-up and medical surveillance in the construction sector, particularly for workers classified as fit with conditions, fit with limitations, or temporarily unfit. Feasibility within operational constraints was a key contributor to the lack of consensus. Rather than prescribing detailed follow-up procedures, the revisions positioned follow-up as an organisational responsibility requiring coordinated involvement of health professionals involved in FFWAH assessment, employers, safety personnel, and line managers, supported by appropriate organisational policy to enable ongoing monitoring, accommodation, and reintegration where indicated.

#### Legal, ethical, and regulatory compliance

Table 8(b) summarises expert feedback and revisions relating to legal, ethical, and regulatory compliance. Consensus was achieved on clarity, relevance, and practicality.

Participants agreed that the principles outlined were broadly adequate to support FFWAH assessment when applied consistently within professional scope. No substantive changes were required; revisions were limited to minor language edits and terminological alignment for internal consistency.

#### Conclusion

Table 9 summarises the expert feedback and revisions related to the conclusion of the consensus statement. Consensus was achieved on both the summary and the recommendations.

**Table 9:** Revisions to Conclusion

Salient Discussion Points	Illustrative Quotes	Revisions Made
The benefit of the consensus statement to workers should be stated.	“… you omit [the benefit to] the most important part, which is the patient-cum-worker-cum-person who comes to work mostly to earn money …”. [P1]	Benefit stated in Conclusion.
Recommendation that professional bodies ensure their professionals meet legal and ethical requirements to perform their respective roles.	[Professional bodies] must look at what’s registered, and within that registration, at every council there’s a scope of practice, and one must look at that scope of practice …” [P1]	Recommendation included that relevant statutory and professional bodies address current gaps in qualification recognition, registration processes, and scope of practice frameworks for professionals working in this field.
Recommendation for policy development and further research.	“… you should put a very strong consideration on doing a separate study, or within your study, on what are the operational demands of people working under a fall risk …”. [P1]	Recommendations included.

Discussion emphasised the importance of clearly articulating the benefits of the consensus statement for workers and of strengthening recommendations on professional accountability. Revisions reinforced a worker-centred framing and highlighted the need for relevant statutory and professional bodies to address identified gaps in qualification recognition, registration processes, and scope of practice frameworks relevant to FFWAH assessment.

## Discussion

This study employed a vMNGT to revise and consolidate a draft interdisciplinary consensus statement on assessing FFWAH in the South African construction industry. Substantial consensus was achieved on 20 of 27 ratings (74%), providing strong support for most sections while identifying areas requiring further clarification or contextual adaptation. The revised consensus statement (Additional file 4) sharpens the scope of application; clarifies key foundational definitions; strengthens guidance on occupational risk exposure and worker–job specification; more explicitly reflects the multifaceted nature of FFWAH assessment, encompassing physical, cognitive, behavioural, environmental, and contextual factors; expands fitness outcomes to include a ‘fit with limitations’ classification; and introduces the concept of a competent, registered, and authorised person to acknowledge the need for appropriate competence without prescriptively assigning responsibility to specific professions.

**Fig. 2:**
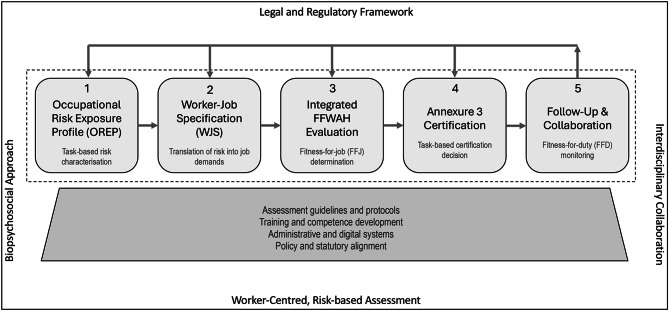
Framework for assessing fitness for work at heights in the South African construction

Figure 2 depicts the framework underpinning the revised consensus statement. The framework conceptualises FFWAH assessment as a five-step process comprising: (1) development of an OREP; (2) translating identified risks into specified job demands through the WJS; (3) an integrated FFWAH evaluation to determine fitness-for-job based on the alignment between worker capacities and job demands; (4) task-based certification of fitness using the Annexure 3 medical certificate; and (5) follow-up and workplace collaboration to support ongoing fitness-for-duty monitoring and risk management over time.

This five-step procedure operates within a set of overarching governance principles that establish the context for its application. These principles include legal and regulatory compliance, interdisciplinary collaboration, and a biopsychosocial, worker-centred, and risk-based approach to fitness for work. The procedure is supported by fundamental enablers—assessment guidelines and protocols, training and competency development, administrative and digital systems, and policy and statutory alignment—which provide the structural foundation necessary for consistent and sustainable implementation across construction environments.

Overall, the findings demonstrate strong interdisciplinary consensus—reflected in high agreement across core sections of the consensus statement and convergent qualitative themes—that FFWAH assessment should move beyond basic medical certification towards a structured, risk-based, and job-specific approach. This aligns with international literature recognising falls from height as a multifactorial risk involving physical, cognitive, psychosocial, behavioural, and environmental factors, which cannot be fully addressed through isolated medical screening alone [4, 17, 49–51]. The explicit utilisation of OREP and WJS as core components directly addresses gaps identified in the Phase 1 scoping review, where inconsistent consideration of task demands, behavioural factors, and changing work environments weakened the consistency and validity of fitness determinations. [15, 23, 25, 52].

Areas where consensus was not achieved provide insight into current limitations of FFWAH practice. Lack of clarity in key definitions, the OREP, and follow-up procedures reflects the absence of shared terminology and standardised guidance across disciplines. Practical concerns, particularly regarding follow-up and surveillance in the construction industry, highlight structural constraints, including temporary worksites, contract-based employment, and limited continuity of occupational health services [53–56]. These constraints suggest that effective FFWAH management requires shared responsibility among health professionals involved in FFWAH assessment, employers, and site-based personnel, rather than reliance on periodic medical assessments alone.

Professional roles and scopes of practice emerged as a recurrent point of discussion. Ambiguity in South African legislation regarding the statutory construct of the so-called occupational health practitioner, together with overlapping and inconsistently regulated involvement of various professionals in FFWAH assessments—including, but not limited to, occupational medical practitioners, occupational health nurses, occupational therapists, and construction health and safety practitioners—contributes to inconsistent practices. The adoption of the term competent, registered, and authorised person within the consensus statement aims to provide legally defensible clarity while avoiding prescriptive allocation of roles in the absence of statutory consensus. This approach highlights the need for broader professional and regulatory engagement to establish a clearer delineation of roles and governance mechanisms.

From a practice and policy perspective, the revised consensus statement offers a transparent framework to support more consistent and defensible FFWAH assessments. For practitioners, it provides structured guidance that explicitly links fitness decisions to job demands and risk exposure. For industry and policymakers, the findings highlight the need for improved regulatory clarity regarding follow-up, medical surveillance, and professional responsibilities, as well as opportunities for system-level interventions such as shared OREP resources, training standards, and national guidelines. Together, these findings suggest that strengthening principles and processes is a necessary precursor to developing validated tests, tools, and fitness thresholds for work at heights.

### Strengths and limitations

This is the first interdisciplinary consensus statement on FFWAH assessment in South Africa, drawing on perspectives from occupational medicine, occupational health nursing, occupational therapy, and construction health and safety. The vMNGT enabled geographically dispersed experts to actively engage in structured discussion, demonstrating the feasibility of virtual platforms for consensus-building research. Transparent documentation of amendments enhances credibility and reproducibility, while integration of the proposed framework provides a practical tool to accompany the consensus statement.

As with other NGT-based studies, the small expert panel limits generalisability beyond the participating disciplines, and the findings reflect the perspectives of the individual experts rather than those of all professionals within those fields. Workers and employers were deliberately excluded to prioritise professional perspectives, which limits insight into feasibility and acceptability in practice. In addition, the consensus statement was not tested in real-world implementation. Further research is needed to evaluate the application in diverse construction contexts, including resource-constrained and informal settings, and to address unresolved issues such as defining operational demands of fallrisk work, developing testing protocols and fitness thresholds, and embedding a biopsychosocial approach aligned with ICF standards [29] into routine practice.

## Conclusion

This study revised and consolidated a draft interdisciplinary consensus statement on assessing FFWAH in the South African construction industry using a vMNGT. The process achieved substantial expert consensus across most components of the statement, while also identifying areas requiring clarification, particularly regarding definitions, occupational risk exposure profiling, and follow-up practices.

Importantly, the revised consensus statement is not presented as a final guideline or prescriptive standard, but as a foundational, principles- and process-based framework. It directly addresses the current absence of standardised FFWAH assessment guidance by articulating shared concepts, assessment logic, and interdisciplinary roles to support more consistent, transparent, and defensible fitness determinations. In doing so, it shifts FFWAH assessment away from reliance on generic medical certification toward a structured, job-specific, and risk-based approach grounded in biopsychosocial principles.

Beyond its contribution to knowledge, the consensus statement is intended to inform practice and policy development. In the short term, it provides a common reference point for professionals involved in determining and managing fitness for work at heights across clinical, functional, and workplace contexts. In the medium term, it offers a platform for professional bodies, regulators, and industry stakeholders to develop aligned guidance, training standards, and governance mechanisms, including clarification of professional roles, follow-up responsibilities, and documentation practices within the existing regulatory framework.

Future work should build on this foundation by translating the agreed principles and processes into operational tools, such as standardised occupational risk exposure profiles, worker–job specification templates, and guidance on fitness classification and follow-up. Validation and feasibility testing in diverse construction contexts, alongside engagement with employers, workers, and policymakers, will be critical to ensure that the framework is practical, equitable, and responsive to real-world constraints. Together, these steps are necessary to support safer, fairer, and more consistent management of fitness for work at heights in the South African construction industry.

## Electronic supplementary material

Below is the link to the electronic supplementary material.

Supplementary material 1

Supplementary material 2

Supplementary material 3

Supplementary material 4

## Data Availability

The datasets generated and analysed during this study are available from the corresponding author on reasonable request.

## Declarations

### Ethial approval

Ethical approval was obtained from the Faculty of Health Sciences Research Ethics Committee, University of Pretoria (Ref. 486/2021). All participants provided written informed consent.

## Abbreviations

CS: Consensus statement
FFD: Fit for duty
FFJ: Fit for job
FFWAH: Fitness for work at heights
ICF: International Classification of Functioning, Disability, and Health
IQR: Interquartile range
OREP: Occupational risk exposure profile
vMNGT: Virtual Modified Nominal Group Technique
WJS: Worker-job specification
