# Supplementary material for: Development of an interdisciplinary consensus statement for assessing fitness for work at heights in the South African construction industry: a virtual Modified Nominal Group Technique study
Source: J Occup Med Toxicol. 2026 Feb 26;21:7. doi: 10.1186/s12995-026-00500-0 (PMC12983645; doi:10.1186/s12995-026-00500-0)
Supplement: Supplementary file 2 — Supplementary material 2 [file 12995_2026_500_MOESM2_ESM.pdf]

LYNDSEY SWART
Ph.D Research
Semi-structured one-on-one interviews with expert panel

Demographics Form

PLEASE PRINT

Name						
Discipline/ content expertise	OMP*	☐	OHNP*	☐	OT*	☐
	CHSP*	☐				
	Special expertise:					
Formal under and postgraduate qualification(s) and year(s) obtained						
Name your professional registrations	HPCSA	☐	SASOM	☐	Saiosh	☐
	SANC	☐	SASOHN	☐	OTOH	☐
	SACPCMP	☐	OTASA	☐		☐
	Other:					
Total years' professional experience:						
Main location(s) of practice	Eastern Cape	☐	KwaZulu-Natal	☐	Northern Cape	☐
	Free State	☐	Limpopo	☐	North West	☐
	Gauteng	☐	Mpumalanga	☐	Western Cape	☐
OMPs, OHNPs, CHSPs complete this section						
Total years experience in construction industry						
Employment setting and sector *(Tick all applicable)*	Self-employed	☐	Mining-related construction	☐	Renewable energy construction	☐
	Small/ medium enterprise	☐	Civil / infrastructure construction	☐	Industrial construction	☐
	Corporate	☐	Building construction	☐		☐
	Other:					
Do you work exclusively in the construction industry?	Yes	☐				
	No	☐				

Demographics Form

OTs complete this section

Years experience in vocational rehabilitation						
Main referral sources *(tick all that apply)*	WCA/ RMA/ FEM		Employer		Insurance company	
	OMP*		DEL*		GP/ medical specialist	
	OHNP*		DOH*			
	Other:					
Vocational rehabilitation services provided *(Tick all that apply)*	Work capacity evaluation		Reasonable accommodation		Disability audits	
	Job analysis		Preventative interventions		Disability aware-ness/ sensitisation	
	Work hardening/ conditioning		Return to work initiatives		Work related skills training	
	Other/ comments:					

* OMP = Occupational medical practitioner
CHSP = Construction Health and safety practitioner.
DEL = Department of Employment and Labour
OHNP = Occupational health nursing practitioner
OT = Occupational therapist
DOH = Department of Health
