# Supplementary material for: Development of an interdisciplinary consensus statement for assessing fitness for work at heights in the South African construction industry: a virtual Modified Nominal Group Technique study
Source: J Occup Med Toxicol. 2026 Feb 26;21:7. doi: 10.1186/s12995-026-00500-0 (PMC12983645; doi:10.1186/s12995-026-00500-0)
Supplement: Supplementary file 3 — Supplementary material 3 [file 12995_2026_500_MOESM3_ESM.pdf]

Evaluation of the Draft Consensus Statement on Fitness for Work at Heights

---

Start of Block: Block 1: Welcome Message

Thank you for participating in this modified Nominal Group Technique (vMNGT) session. This survey provides a structured opportunity to evaluate each section of the Draft Consensus Statement on the Assessment of Fitness for Work at Heights (FFWAH) in the South African Construction Industry. Please have your copy of the Draft Consensus Statement available while completing this survey. Your responses will be anonymised and cannot be linked back to you. Only summary results will be shared, and no identifying information will be included.

End of Block: Block 1: Welcome Message

Start of Block: Block 2: General Instructions

Instructions For each section of the Draft Consensus Statement, you will assess aspects such as clarity, relevance, and feasibility. Please note that some sections may have slightly different evaluation aspects, which will be clearly indicated with each question. **Use the following 5-point Likert scale:** 1 = Strongly Disagree 2 = Disagree 3 = Neutral (neither agree nor disagree) 4 = Agree 5 = Strongly Agree Rate each section using your professional judgment and the discussions already held. If you select 1 = Strongly Disagree or 2 = Disagree, **please provide a brief comment explaining your concern and, where possible, suggest improvements in the text box below**. Comments are optional for other ratings, but are always welcome. Select 3 = Neutral only if you genuinely feel unable to agree or disagree. Please complete the evaluation independently — no discussion during this step. You must complete all ratings in a section before the survey will allow you to move on to the next section. Please ensure you have considered the **entire** section of the Consensus Statement before completing the ratings. Once you proceed to the next section, you will not be able to return or make any changes.

End of Block: Block 2: General Instructions

Start of Block: Block 3: Section 1–Introduction

Please refer to ***Section 1: Introduction*** on your Draft Consensus Statement. Please rate the following aspects of the ***Introduction***:

	1 = Strongly Disagree (6)	2 = Disagree (7)	3 = Neutral (9)	4 = Agree (11)	5 = Strongly Agree (12)
The **purpose, scope, and relevance** of the Consensus Statement is clearly outlined. (1)	○	○	○	○	○
The **credibility** of the Consensus Statement **development process** is adequately established. (2)	○	○	○	○	○

Optional: Please provide any comments or suggestions for improving the ***Introduction***.

____

____

____

____

____

End of Block: Block 3: Section 1–Introduction

Start of Block: Block 4: Section 2–Key Definitions

Please refer to ***Section 2: Key Definitions*** on your Draft Consensus Statement. Please rate the following aspects of the ***Key Definitions***:

	1 = Strongly Disagree (6)	2 = Disagree (7)	3 = Neutral (9)	4 = Agree (11)	5 = Strongly Agree (12)
The key definitions are **clear, accurate, and easy to understand**. (1)	○	○	○	○	○
The key definitions are **relevant** to FFWAH evaluation (2)	○	○	○	○	○

Optional: Please provide any comments or suggestions for improving the ***Key*** Definitions section***.***

____

____

____

____

____

End of Block: Block 4: Section 2–Key Definitions

Start of Block: Block 5: Section 3.1–5-step procedure for Assessing FFWAH

Please refer to ***Section 3.1: Five-step Procedure for Assessing FFWAH*** on your Draft Consensus Statement. Please rate the following aspects of **Section 3.1**:

	1 = Strongly Disagree (1)	2 = Disagree (2)	3 = Neutral (3)	4 = Agree (4)	5 = Strongly Agree (5)
The 5-step process is **clear and comprehensible**. (1)	○	○	○	○	○
The 5-step process **provides a robust framework** for assessing FFWAH. (2)	○	○	○	○	○
The 5-step process is **practical and feasible** for use in the South African construction industry (3)	○	○	○	○	○

Optional: Please provide any comments or suggestions for improving the ***Pr***ocedure for Assessing FFWAH section.

____

____

____

____

____

End of Block: Block 5: Section 3.1–5-step procedure for Assessing FFWAH

Start of Block: Block 6: Section 3.2–Occupational Risk Exposure Profile (OREP)

Please refer to ***Section 3.2: Occupational Risk Exposure Profile (OREP)*** on your Draft Consensus Statement. Please rate the following aspects of **Section 3.2**:

	1 = Strongly Disagree (1)	2 = Disagree (2)	3 = Neutral (3)	4 = Agree (4)	5 = Strongly Agree (5)
The OREP section is **clear and comprehensible**. (1)	○	○	○	○	○
The OREP section reflects **important principles** for FFWAH assessment. (2)	○	○	○	○	○
The OREP statements are **practical and feasible** for use in the South African construction industry. (3)	○	○	○	○	○

Optional: Please provide any comments or suggestions for improving the ***OREP*** section.

____

____

____

____

____

End of Block: Block 6: Section 3.2–Occupational Risk Exposure Profile (OREP)

Start of Block: Block 7: Section 3.3–Worker-Job-Specification (WJS)

Please refer to ***Section 3.3: Worker-Job-Specification (WJS)*** on your Draft Consensus Statement.   Please rate the following aspects of section 3.3:

	1 = Strongly Disagree (1)	2 = Disagree (2)	3 = Neutral (3)	4 = Agree (4)	5 = Strongly Agree (5)
The WJS section is **clear and comprehensible**. (1)	○	○	○	○	○
The WJS section reflects **important principles** for FFWAH assessment. (2)	○	○	○	○	○
The WJS statements are **practical and feasible** for use in the South African construction industry. (3)	○	○	○	○	○

Optional: Please provide any comments or suggestions for improving the ***WJS*** section.

____

____

____

____

____

End of Block: Block 7: Section 3.3–Worker-Job-Specification (WJS)

Start of Block: Block 8: Section 3.4–Fitness for work-at-height (FFWAH) evaluation

Please refer to ***Section 3.4: Fitness for work-at-height (FFWAH) Evaluation*** on your Draft Consensus Statement.   Please rate the following aspects of section 3.4:

	1 = Strongly Disagree (1)	2 = Disagree (2)	3 = Neutral (3)	4 = Agree (4)	5 = Strongly Agree (5)
The FFWAH evaluation section is **clear and comprehensible**. (1)	○	○	○	○	○
The FFWAH evaluation section reflects **important principles** for FFWAH assessment. (2)	○	○	○	○	○
The FFWAH evaluation statements are **practical and feasible** for use in the South African construction industry. (3)	○	○	○	○	○

Optional: Please provide any comments or suggestions for improving the ***FFWAH Evaluation*** section.

____

____

____

____

____

End of Block: Block 8: Section 3.4–Fitness for work-at-height (FFWAH) evaluation

---

Start of Block: Block 9: Section 3.5–Annexure 3 Medical Certificate of fitness

Please refer to ***Section 3.5: Annexure 3 Medical Certificate of Fitness (Annexure 3)*** on your Draft Consensus Statement. Please rate the following aspects of section 3.5:

	1 = Strongly Disagree (1)	2 = Disagree (2)	3 = Neutral (3)	4 = Agree (4)	5 = Strongly Agree (5)
The Annexure 3 section is **clear and comprehensible**. (1)	○	○	○	○	○
The Annexure 3 section reflects **important principles** for FFWAH assessment. (2)	○	○	○	○	○
The Annexure 3 statements are **practical and feasible** for use in the South African construction industry. (3)	○	○	○	○	○

Optional: Please provide any comments or suggestions for improving the ***Annexure 3 Medical Certificate of Fitness*** section.

____

____

____

____

____

End of Block: Block 9: Section 3.5–Annexure 3 Medical Certificate of fitness

Start of Block: Block 10: Section 3.6–Follow-up of workers with limitations or restrictions.

Please refer to ***Section 3.6: Follow-up of Workers with Limitations or Restrictions*** on your Draft Consensus Statement. Please rate the following aspects of section 3.6:

	1 = Strongly Disagree (1)	2 = Disagree (2)	3 = Neutral (3)	4 = Agree (4)	5 = Strongly Agree (5)
The Follow-up of Workers section is **clear and comprehensible**. (1)	○	○	○	○	○
The Follow-up of Workers section reflects **important principles** for FFWAH assessment. (2)	○	○	○	○	○
The Follow-up of Workers statements are **practical and feasible** for use in the South African construction industry. (3)	○	○	○	○	○

Optional: Please provide any comments or suggestions for improving the ***Follow-up of Workers with Limitations or Restrictions*** section.

____

____

____

____

____

End of Block: Block 10: Section 3.6–Follow-up of workers with limitations or restrictions.

Start of Block: Block 11: Section 3.7–Legal, ethical and regulatory compliance

Please refer to ***Section 3.7: Legal, Ethical and Regulatory Compliance*** on your Draft Consensus Statement.   Please rate the following aspects of section 3.7:

	1 = Strongly Disagree (1)	2 = Disagree (2)	3 = Neutral (3)	4 = Agree (4)	5 = Strongly Agree (5)
The Legal, Ethical & Regulatory Compliance section is **clear and comprehensible**. (1)	○	○	○	○	○
The Legal, Ethical & Regulatory Compliance section reflects **important principles** for FFWAH assessment. (2)	○	○	○	○	○
The Legal, Ethical & Regulatory Compliance statements are **practical and feasible** for use in the South African construction industry. (3)	○	○	○	○	○

Optional: Please provide any comments or suggestions for improving the ***Legal, Ethical and Regulatory Compliance*** section.

____

____

____

____

____

End of Block: Block 11: Section 3.7–Legal, ethical and regulatory compliance

Start of Block: Block 12: Section 4–Conclusion

Please refer to ***Section 4: Conclusion*** on your Draft Consensus Statement. Please rate the following aspects of **Section 4**:

	1 = Strongly Disagree (1)	2 = Disagree (2)	3 = Neutral (3)	4 = Agree (4)	5 = Strongly Agree (5)
Clearly summarises the **key outcomes and implications** for occupational health practice (1)	o	o	o	o	o
Provides **appropriate recommendations** for future action or evaluation. (2)	o	o	o	o	o

Optional: Please provide any comments or suggestions for improving the ***Conclusion*** section.

____

____

____

____

____

End of Block: Block 12: Section 4–Conclusion
