# Supplementary material for: Development of an interdisciplinary consensus statement for assessing fitness for work at heights in the South African construction industry: a virtual Modified Nominal Group Technique study
Source: J Occup Med Toxicol. 2026 Feb 26;21:7. doi: 10.1186/s12995-026-00500-0 (PMC12983645; doi:10.1186/s12995-026-00500-0)
Supplement: Supplementary file 4 — Supplementary material 4 [file 12995_2026_500_MOESM4_ESM.pdf]

Consensus Statement on Assessing Fitness for Work at Heights in the South African Construction Industry

1. Introduction

The safe performance of work at heights is a critical occupational health and safety priority in the South African construction industry. The Construction Regulations, 2014, promulgated under the Occupational Health and Safety Act 85 of 1993 (OHSA), require workers performing tasks at heights to possess a valid medical certificate of fitness; however, there is limited formal guidance on how such fitness should be assessed. This has resulted in ambiguity and inconsistency in the practical implementation of fitness-for-work-at-height (FFWAH) practices across the industry, particularly regarding assessment roles, standards, and procedures.

This consensus statement addresses these challenges by proposing a structured, evidence-based framework for assessing FFWAH. It defines key concepts, outlines a five-step assessment process, and establishes broad principles to support consistent, legally defensible, and ethically sound practices. This consensus statement represents a foundational step toward standardising FFWAH assessments in South Africa's construction sector. It aims to assist occupational health practitioners, employers, safety professionals, and policymakers in fostering safer work environments by promoting shared terminology, enhancing regulatory alignment, and strengthening interdisciplinary collaboration. Beyond immediate application, the statement provides a practical and adaptable platform for developing more detailed, context-specific guidance, thereby contributing to improved assessment practices and to the broader field of occupational health systems research.

The statement focuses specifically on the assessment of fitness for job (FFJ), which is distinct from fitness for duty (FFD), a day-to-day determination typically made by the employer or supervisor.

Throughout this Consensus Statement, the term "competent, registered, and authorised person" is used to describe individuals responsible for conducting or contributing to FFWAH assessments. This inclusive terminology is designed to remain profession-neutral while aligning with South African legal and regulatory requirements.[1] By using this phrase—defined in detail under *Key Definitions*—the Statement supports interdisciplinary application without prescribing specific professional roles, while ensuring accountability, ethical standards, and compliance are upheld.

The consensus statement was developed through a rigorous multi-phase process, including a systematic scoping review of available evidence[1], an exploratory qualitative study with interdisciplinary experts (occupational medical practitioners, occupational health nurses, occupational therapists, and construction health and safety practitioners), and a structured consensus development phase using a virtual modified Nominal Group Technique. Ethical approval for the development of this statement was granted by the Research Ethics Committee, Faculty of Health Sciences, University of Pretoria (Ref. 486/2021).

For the purposes of this consensus statement, the assessment of FFWAH refers to an integrated medical and functional evaluation of a worker's ability to safely perform all aspects of the job. This includes accessing and egressing the work area, managing the physical and psychological demands of working at height, and performing specific tasks while in elevated positions. Central to this is the ability to tolerate fall risk. The assessment must also consider the worker's capacity to endure emergency rescue procedures, such as suspension in a harness following a fall, which may carry additional physiological risks. Beyond fall-related hazards, the evaluation should reflect a holistic understanding of the job's overall demands. This includes physical, cognitive, psychosocial, and environmental factors, consistent with internationally recognised biopsychosocial frameworks, such as the World Health Organisation's International Classification of Functioning, Disability and Health (ICF).[2]

Abbreviations

FFD	Fitness for duty
FFJ	Fitness for job
FFWAH	Fitness for work at heights
FJDA	Functional job demands analysis
HPCSA	Health Professions Council of South Africa
ICF	International Classification of Functioning, Disability and Health
JSFE	Job-specific functional evaluation
OHSA	Occupational Health and Safety Act (1993
SACPCMP	South African Council for the Project and Construction Management Professions
SANC	South African Nursing Council
WAH	Work at heights

2. Key Definitions

Competent, registered, and authorised person is defined as:

a. **Competent**: This refers to the definition of a *"competent person"* as outlined in various regulations under the OHSA and further clarified in the Department of Employment and Labour's Competent Person Guideline.[3] It implies that the individual possesses the required knowledge, training, experience, and qualifications to perform the task safely and effectively.
b. **Registered**: This indicates that the individual is statutorily registered in terms of relevant Acts of Parliament and is practising within the scope of practice defined by their professional regulatory body (e.g., HPCSA, SANC, SACPCMP).
c. **Authorised**: In terms of Sections 8 and 9 of the OHSA, the responsibility for occupational health measures, including FFWAH assessments, ultimately resides with the employer. Therefore, the employer is required to identify and formally appoint a person who is both competent and legally eligible to perform these duties.

Fall risk: any potential exposure to falling either from, off, or into. (Construction Regulations, 2014)

Fit for duty (FFD): A worker's day-to-day readiness to perform their job safely and without undue risk. FFD is about ensuring a worker is safe to work on a given day, rather than assessing their overall health. It is typically monitored by the employer, supervisor, or designated health and safety personnel, and is distinct from fitness for job (FFJ), which involves structured medical and functional assessments.

Fit for job (FFJ): A worker's medical and functional ability to safely meet the physical, cognitive, psychosocial, and environmental risks and demands of a specific job, in line with applicable health and safety standards. This determination considers the full scope of the job, including the associated risks and exposures, as identified in the Occupational Risk Exposure Profile (OREP) and the Worker Job Specification (WJS).

Functional Job Demands Analysis (FJDA): A structured, task-level assessment that identifies and quantifies the physical, cognitive, psychosocial, and environmental demands of a specific job. It captures detailed functional requirements, including strength, coordination, attention, and communication. Unlike the OREP, which focuses on job-related hazards and exposures, the FJDA analyses what the job requires of the worker and is used to supplement or elaborate on the Worker-Job Specification (WJS) where greater task-level specificity is required.

Job-specific functional evaluation (JSFE): A structured, performance-based assessment that evaluates a worker's ability to safely and effectively perform the essential functions of a specific job. Guided by the OREP and the Worker-Job Specification (WJS), the JSFE considers the worker's physical, cognitive, and psychosocial capacities in the context of the job's functional demands. It reflects a holistic model of functioning that integrates health conditions, activity limitations, participation constraints, and environmental or organisational factors, in line with the principles of the ICF.

Work at heights (WAH): Work in any place where, if precautions were not taken, the worker is exposed to a fall risk that can cause personal injury. (Work at Height Regulations, 2005 [UK])

3. Consensus Statement

3.1. Five-Step procedure for assessing FFWAH

a. A structured five-step procedure is recommended for assessing FFWAH, providing a consistent framework that is comprehensive, job-related, and legally defensible:
   - Step 1. Compile an OREP.
   - Step 2. Develop a Worker-Job Specification (WJS).
   - Step 3. Conduct the FFWAH evaluation.
   - Step 4. Issue the medical certificate of fitness in the form of Annexure 3 (Construction Regulations, 2014).
   - Step 5. Follow up and monitoring of workers with restrictions, limitations, or conditional clearance.

3.2. Occupational risk exposure profile (OREP)

a. The employer is responsible for ensuring that a comprehensive Occupational Risk Exposure Profile (OREP) is compiled for each work-at-height task or role. The OREP must be developed by a competent, registered, and authorised person with appropriate training and knowledge of the job's operational requirements and associated hazards. It serves as the foundational document for determining fitness-for-work requirements and must be kept current and specific to the task, work environment, and operational conditions.

b. The OREP is a job-specific profile that identifies and characterises the inherent hazards, environmental exposures, and operational demands associated with work at height. It must consider the full task cycle, including:
   i. Access and egress to and from the elevated work area
   ii. The performance of job-specific tasks while at height
   iii. The fall risk and related exposures during all phases of the task

c. The OREP should explicitly capture the physical, cognitive, psychosocial, behavioural, and environmental demands of the role that may impact worker health, functioning, and safety.

d. Each OREP must also:
    i. Describe the type, intensity, frequency, and duration of all exposures
    ii. Detail the existing control measures and emergency arrangements, including requirements under the site-specific Fall Protection Plan
    iii. Align with relevant statutory regulations and industry standards, including the Construction Regulations (2014)

e. The OREP must be made available to the occupational health professionals responsible for conducting medical surveillance and fitness-for-work assessments. It provides the risk-informed foundation for:
    i. Developing the Worker-Job Specification (WJS); and
    ii. Conducting legally defensible, job-specific FFWAH evaluations.

f. The employer must ensure that a centralised and accessible database of OREPs is maintained to support consistency, currency, and cross-role integration of fitness assessment procedures.

3.3. Worker-job-specification (WJS)

a. The Worker-Job Specification (WJS) is a structured, job-specific profile developed by a competent, registered, and authorised health professional, based on the Occupational Risk Exposure Profile (OREP). It outlines the essential requirements for safe and effective job performance across the physical, sensory, cognitive, psychosocial, behavioural, and environmental domains. The WJS defines the minimum health standards needed for work at height and identifies potential worker-related risks that may compromise safe performance.

b. The WJS serves as a key reference in the FFWAH assessment process. It:
    i. Informs the selection of appropriate assessment methods—medical, clinical, functional, psychological, and contextual;
    ii. Establishes testing protocols and reference thresholds aligned with legal and regulatory requirements;
    iii. Supports consistent, evidence-based, and defensible decisions regarding job placement, conditional clearance, and the application of limitations or restrictions.

c. Where the OREP does not sufficiently detail the functional demands of a job, a Functional Job Demands Analysis (FJDA) may be conducted by a competent, registered, and authorised person. The FJDA provides a task-level assessment of

the actual functional requirements of the role, supporting fair, practical, and legally aligned fitness determinations.

d. The WJS must be developed and applied in accordance with relevant legal frameworks, industry standards, and professional scopes of practice to ensure both regulatory compliance and the ethical management of workers' health and fitness.

3.4. FFWAH evaluation

a. The FFWAH evaluation is a structured, job-specific assessment conducted by a competent, registered, and authorised person to determine whether a worker can safely perform work at height. It is based on the Worker-Job Specification (WJS) and the OREP, considering a range of factors, including the worker's medical status, functional ability, cognitive and psychosocial capacity, and the specific demands and context of the job.

b. The evaluation begins with a job-specific medical screening. Where indicated, this may be followed by additional targeted medical investigations or functional assessments relevant to the task, job setting, or identified risks.

c. Worker fitness is classified as follows:*
   i. **Unconditionally Fit** – The worker meets all medical, functional, cognitive, and psychosocial job requirements without any identified risks or need for control measures or accommodations.
   ii. **Fit with Conditions** (clearance with controls) – The worker is fit to perform the job, provided specific control measures or accommodations are implemented, such as supervision, assistive devices, modified exposure, or periodic review. These measures are typically time-bound, situational, or reversible, and should be clearly documented and reviewed periodically.
   iii. **Fit with Limitations** (capacities with thresholds) – The worker has a reduced but acceptable capacity in one or more domains. They may safely perform essential job tasks within defined limits – e.g., lifting limited to 15 kg occasionally, or visual acuity within a corrected range. Limitations describe what the worker can safely do, rather than what they cannot.
   iv. **Fit with Restrictions** (activities prohibited) – The worker may safely perform some aspects of the job, but specific tasks or exposures are

explicitly prohibited because they pose unacceptable health or safety risks. Restrictions should be task-specific, clearly stated, and based on documented medical or risk evidence.

v. **Unfit – review if indicated** – The worker does not currently meet the essential medical, functional, cognitive, or psychosocial requirements for safe work at height and cannot perform the role safely, even with reasonable accommodation. Where the condition is potentially reversible or reviewable, specify a review date or criteria for reassessment. If no improvement is anticipated, note: “Long-term unfitness anticipated – further management or redeployment recommended.

d. This classification system supports consistent and transparent decision-making and should always be guided by the Worker-Job Specification and the Occupational Risk Exposure Profile.

* ***Note**: All classifications are made within the context of the Construction Regulations, and do not exempt the employer from implementing fall protection measures as part of the site-specific Fall Protection Plan.*

3.5. Annexure 3: certificate of fitness

a. The certificate of fitness, issued in the form of Annexure 3 by a competent, registered, and authorised person, must be job-specific and clearly indicate the job title, the corresponding OREP, the fitness classification (as outlined in 3.4.c), and the validity period. It must also specify any applicable conditions, limitations, or restrictions, as well as any required accommodations, monitoring, reassessment intervals, or other interventions necessary to ensure safe work performance.

3.6. Follow-up of workers

a. Follow-up and monitoring of workers classified as fit with restrictions, limitations, or conditional clearance must be carried out by a competent, registered, and authorised person.

b. That professional must work in collaboration with the employer, relevant safety personnel, and line management to manage these workers effectively, following fitness for duty principles. This includes implementing the specified conditions, limitations, or restrictions; providing appropriate accommodations and workplace

adjustments; overseeing medical or rehabilitative monitoring as needed; and arranging any necessary periodic re-evaluations to support day-to-day determinations of worker readiness to perform their duties safely and without undue risk.

3.7. Legal, ethical and regulatory compliance

a. Only competent, registered, and authorised persons should perform FFWAH assessments in accordance with applicable laws, regulations, and professional scopes of practice.

b. All assessments must maintain strict confidentiality and uphold ethical principles, ensuring that workers are treated with dignity and fairness.

c. Any decisions that may affect a worker's employment status must be communicated transparently and addressed through collaborative planning involving all relevant parties, including the health professionals responsible for the FFWAH assessment and follow-up, the employer, and the worker.

d. Workers have a duty to actively participate in and cooperate with the FFWAH assessment process.

4. Conclusion

This consensus statement provides a structured, interdisciplinary framework for assessing FFWAH in the South African construction industry. It defines essential concepts, outlines a practical and legally compliant five-step process, and integrates key principles of sound occupational health practice.

The adoption of this framework aims to improve the consistency, transparency, and fairness of fitness-to-work assessments, thereby encouraging safer work environments and enhanced regulatory compliance. It offers guidance for occupational health professionals, employers, and industry stakeholders, while also safeguarding the rights and safety of workers by promoting equitable, job-specific assessments that are both evidence-informed and defensible.

To better define roles and responsibilities in assessing and managing FFWAH, it is vital that relevant statutory and professional bodies address current gaps in qualification recognition,

registration processes, and scope of practice frameworks for professionals in this field, ensuring compliance with legal and ethical requirements.

The consensus statement should act as a foundation for ongoing research and policy development in this field. Research priorities include understanding the operational demands of work under fall-risk conditions and establishing testing protocols and fitness reference thresholds that meet legal and regulatory standards. Policy development is equally important for guiding the follow-up of workers assessed as unfit, conditionally fit, fit with restrictions, or fit with limitations, and for supporting the integration of a biopsychosocial approach into fitness-for-work assessments. Pursuing research and policy development in a coordinated and context-sensitive manner will help ensure that implementation is evidence-based and practically relevant. Continuous evaluation of the framework's application in real-world settings is recommended to maintain its relevance and encourage ongoing improvement in assessing FFWAH.

January 2026

References

1. Swart L, Buys T, Claassen N. Mapping the evidence on the assessment of fitness to work at heights: a scoping review. BMJ Open. 2025;15:93525.
2. World Health Organization. International Classification of Functioning, Disability and Health (ICF) [Internet]. Geneva: World Health Organization (WHO); 2001 [cited 2025 Jul 5]. Available from: https://www.who.int/standards/classifications/international-classification-of-functioning-disability-and-health
3. Department of Employment and Labour. Competent Person Guideline [Internet]. [cited 2023 Jul 9]. Available from: https://cdn.ymaws.com/www.saiosh.co.za/resource/resmgr/docs/2021_folder_2/competent_person_guidelines_.pdf
